# Rapid Detection of *Cysticercus cellulosae* by an Up-Converting Phosphor Technology-Based Lateral-Flow Assay

**DOI:** 10.3389/fcimb.2021.762472

**Published:** 2021-11-10

**Authors:** Dejia Zhang, Yu Qi, Yaxuan Cui, Weiyi Song, Xinrui Wang, Mingyuan Liu, Xuepeng Cai, Xuenong Luo, Xiaolei Liu, Shumin Sun

**Affiliations:** ^1^ College of Animal Science and Technology, Inner Mongolia University for Nationalities, Tongliao, China; ^2^ Key Laboratory of Zoonosis Research, Ministry of Education, Institute of Zoonosis/College of Veterinary Medicine, Jilin University, Changchun, China; ^3^ Key Laboratory of Veterinary Parasitology of Gansu Province, State Key Laboratory of Veterinary Etioloical Biology, Lanzhou Veterinary Research Institute, Chinese Academy of Agricuitural Sciences, Lanzhou, China; ^4^ College of Veterinary Medicine, Yunnan Agricultural University, Kunming, China

**Keywords:** *Taenia solium*, *Cysticercus cellulosae*, antigen, immune diagnosis, UPT-LF

## Abstract

Cysticercosis is a neglected tropical disease caused by the larvae of *Taenia solium* in pigs and humans. The current diagnosis of porcine cysticercosis is difficult, and traditional pathological tests cannot meet the needs of detection. This study established a UPT-LF assay for the detection of *Cysticercus cellulosae*. UCP particles were bound to two antigens, TSOL18 and GP50; samples were captured, and the signal from the UCP particles was converted into a detectable signal for analysis using a biosensor. Compared to ELISA, UPT-LF has higher sensitivity and specificity, with a sensitivity of 93.59% and 97.44%, respectively, in the case of TSOL18 and GP50 antigens and a specificity of 100% for both. Given its rapidness, small volume, high sensitivity and specificity, and good stability and reproducibility, this method could be used in the diagnosis of cysticercosis.

## Introduction

Cysticercosis is an important zoonotic parasitic disease, which is mainly caused by eating *Taenia solium* larvae by mistake. (Bizhani et al., 2020). This disease is distributed worldwide and is widely prevalent in some relatively backward countries and regions, such as Africa, Asia, and Latin America (Hamamoto et al., 2020). Because some rural areas in relatively backward countries meet the conditions required for the life cycle of Cysticercus, where has poor sanitary conditions, limited or absent meat inspection, low awareness, and inadequate facilities for safe food preparation, which result in accidental ingestion of eggs or larvae containing infective worms and lead to the appearance of cysts in pig muscle or in the human brain and spinal cord, caused serious harm to the economy and human health (Lightowlers et al., 2016; Lightowlers, 2020). From a global perspective, *Cysticercus cellulosae* (*C. cellulosae*) is listed as “one of the Top Ten Foodborne Parasites Harmful to Humans” by the WHO and is also known as “one of the 17 Neglected Tropical Diseases” and “one of the Key Parasitic Diseases Planned and Prevented by the Ministry of Health of the People’s Republic of China”. Therefore, the diagnosis of *C. cellulosae* is particularly essential (Tsai et al., 2013). To reduce the global burden of *C. cellulosae*, serologic tests are commonly used in endemic areas to screen for the parasite and control disease progression by using a combination of antigens and antibodies (Gomez-Puerta et al., 2019). Among the currently used methods for detecting cystic larvae, the most effective serological method is enzyme immunoelectrotransfer blot (EITB). Although EITB is the reference standard and has high sensitivity and specificity, it is unsuitable for widespread use or field testing (Garcia et al., 2018; Romo et al., 2020).

TSOL18 is an oncosphere-stage protein with high protective and immunogenic properties, and GP50 is a cystic larval-stage protein with specificity and immunogenicity. Both are regarded as antigens for the early diagnosis of cysticercosis (Gomez-Puerta et al., 2019). In recent years, up-converting phosphor technology-based lateral flow (UPT-LF) has been increasingly used for the detection of parasitic diseases (Corstjens et al., 2014). In this study, up-converting phosphor (UCP) particles were bound to two antigens, TSOL18 and GP50; samples were captured; and the signal from the UCP particles was converted into a detectable signal for analysis using a biosensor (Liu et al., 2016; Yang et al., 2017).

This study has established a convenient, rapid, specific, stable, and environmentally friendly method for the quantitative detection of *C. cellulosae* at a wide range of concentrations to overcome the shortcomings of current detection methods. The simplicity of the procedure allows detection without professional technicians on site, and the method is suitable for large-scale detection as well as rapid on-site detection.

## Materials and Methods

### Materials

UCP nanomaterials (NaYF4:Yb^3+^, Er^3+^) were purchased from Shanghai SunLipo NanoTech. Nitrocellulose film and glass fiber were purchased from Millipore Company in the United States. Absorbent paper and viscous backing were purchased from Shanghai Jieyi Biotechnology Company. The plastic shell of the test strip was designed and manufactured by Shenzhen Jincanhua Company. Goat anti-pig IgG was purchased from Beijing Baiaolaibo Technology Co., Ltd., and rabbit anti-goat IgG was purchased from Shanghai Absin Bioscience Inc. The instrument used to read the ELISA results was a full-wavelength enzyme labeling instrument (BioTek Instruments, Inc.). The UPT biosensor (UPT-3A-1200) was purchased from Beijing Hotgen Biotechnology Co., Ltd.

### Sample Collection

In this study, positive sera from a pig with *Cysticercus cellulosae* infection were acquired and preserved in the clinical laboratory of the School of Animal Science and Technology, Inner Mongolia University for Nationalities, and sera positive for *Taenia asiatica*, *Toxoplasma gondii*, *Clonorchis sinensis*, and *Trichinella spiralis* were provided by the Institute of Human and Veterinary Diseases, Jilin University.

### Enzyme-Linked Immunosorbent Assay

After diluting the target protein (TSOL18 or GP50) to 1 μg/mL with buffer, then 100 μL antigen was added to each well of the 96-well ELISA plate and incubated overnight at 4°C. After the buffer was absorbed in the 96-well enzyme plate, 150 μL PBST buffer was added to each well, was blotted with clean filter papers 5 times to remove the remaining liquid. Blocking buffer containing 150 μL of 1% BSA was added to each well for sealing, and the plate was washed with PBST buffer 5 times after incubation at 37°C for 2 h. The serum to be tested was diluted with 1% BSA and added to a 96-well enzyme reaction plate. One hundred microliters of the tested serum were added per well, and 3 replicates were performed. Uninfected pig serum was used as the negative control serum and washed 5 times after being placed in a 37°C incubator for 1 h. Goat anti-porcine IgG antibodies labeled with horseradish peroxidase were diluted with blocking buffer at a ratio of 1:2000. Then, 100 μL of diluted antibodies was added to each well, and the plate was washed 5 times after being placed in an incubator at 37°C for 1 h. TMB (100 μL) was added to each well, and the plate was incubated for 15 min at room temperature and in the dark. Then, 50 μL of 2 mol/L H_2_SO_4_ termination solution was added per well, and the reaction was stopped. The absorption value of each pore was detected at a wavelength of 450 nm by ELISA enzyme labeling, and the result was judged. When the value of the sample to be tested was more than 2.1 times the negative control value, it was judged to be positive.

### Preparation and Modification of UCP Particles

The UCP particles in this study are based on sodium fluoride (Na F), which is doped with three rare earth metal elements—yttrium (Y), ytterbium (Yb), and erbium (Er)—to form the crystal structure Na YF_4_:Yb^3+^, Er^3+^, i.e., UCP particles. Although the particles have the unique optical properties of uptransfer luminescence materials, they do not have the ability to bind with biologically active molecules and still need to be processed through surface modification and activation of the particles. This process makes the particles suitable for biological applications. Using ethyl orthosilicate (TEOS), the surface of the UCP particles can be silicified through a series of chemical reactions, resulting in a large number of surface-active groups (Huang Y. et al., 2019). Through these free active groups, UCP particles can be covalently combined with antigens, antibodies, nucleic acids, biotin, and other bioactive molecules to make them biologically active. At the same time, the covalent binding of UCP particles to bioactive molecules makes the binding between the two more secure and ensures that detection is not easily affected by complex samples, laying a solid foundation for the widespread application of UCP-LF detection technology.

### Assembly of the UPT-LF Test Card

The absorbent pad, NC membrane, conjugated pad, and sample pad were placed on the adhesive lamination card to make a complete test card, which was cut transversely into 4 mm wide strips using a high-speed CNC chopping machine, put into a plastic casing, and stored in a dry cabinet for later use. Antigen at a concentration of 0.5 mg/mL and rabbit anti-goat IgG at a concentration of 0.5 mg/mL were then sprayed onto the test strip T and quality control strip C, respectively. The silicified and carboxyl-modified UCP-NPs were centrifuged, and the supernatant was discarded, resuspended in UCP storage buffer, and sonicated 3 times. After vortex sonication, the sample was mixed with 100 μL of buffer (0.01 M phosphate buffer PBS, pH 7.2, containing 1% (w/v) BSA, 10% (w/v) sucrose, and 1% (v/v) Tween-20) and added to the wells of the sample paper. The results were read using an uprotation luminescent biosensor after 15 min of resting. The sensor was used to determine the peak area of detection and the quality control bands, and the detection/quality control band values were used as the final results.

### Sensitivity, Specificity, and Stability Test of the UPT-LF Assay

For evaluation of the sensitivity, the sera of all pigs containing *C. cellulosae* were diluted according to a certain proportion and tested using the UPT-LF assay. Several positive sera were detected by UPT-LF to evaluate the specificity, including sera containing *T. asiatica*, *T. gondii*, *C. sinensis*, and *T. spiralis*. The test results (Vt/Vc value) were recorded at several time points and two temperatures (4°C and 25°C) after sample addition was completed. Using the same batch, three test strips were used for positive and negative serum samples. Each sample was tested three times, the results of the test card at different time points were compared with those detected at 15 min, and the relative deviation (δ) was calculated. δ < 15% was the acceptable standard.

### Data Analysis

The test card was inserted into the UPT-3A-1200 biosensor, the measurement button was pressed, and the test results were obtained in approximately five seconds. The peak areas of the T and C lines were calculated by the special software of the reader, the value was input into Excel, and then the value of the T line was divided by the value of the C line for each test strip. The Vt/Vc value can be used for data analysis.

## Results

### Detection System of the UPT-LF Assay

**Figure 1:**
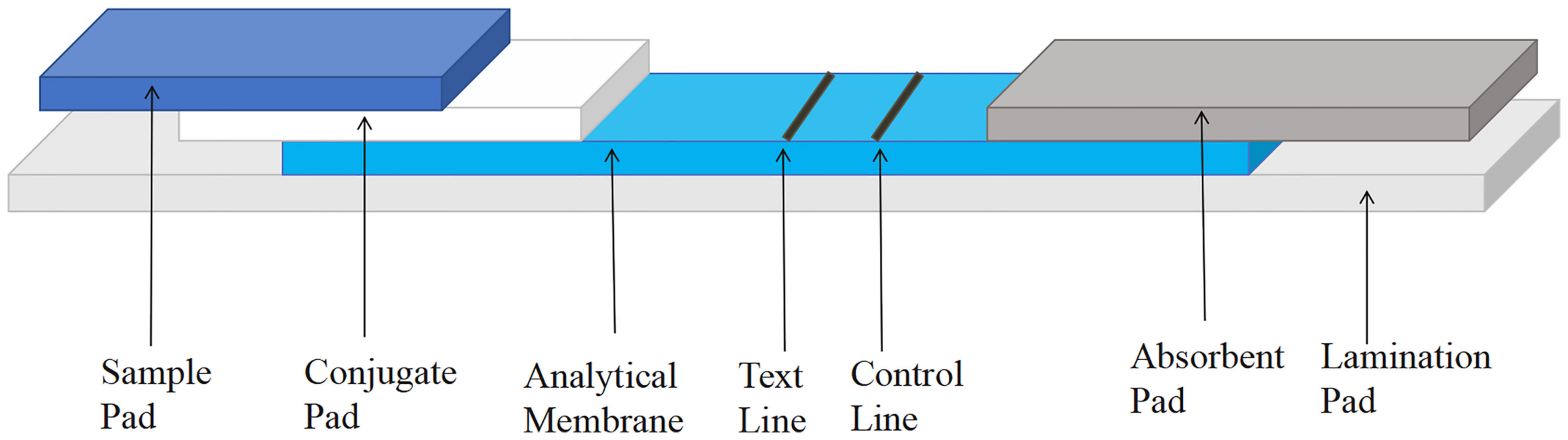
Schematic diagram of the up-converting phosphor technology-based lateral-flow (UPT-LF) strip. The sample flow direction from sample pad, conjugate pad, analytical membrane to the absorbent pad, which all the structures above are on laminating card. The results were obtained by scanning the test card of the UPT-3A-1200 biosensor.

The UPT-LF assay system consists of a sample dilutant, a test card, and a biosensor (**Figure 1**). The test card consists of two parts: the outer shell and the internal test strip. The housing consists of an upper and bottom shell. The upper shell has two windows, followed by a sample addition window and a sample scanning window. The test strips are placed in the grooves of the bottom shell. The portable UPT-3A-1200 biosensor has the advantages of simple operation, rapid detection, high sensitivity, and safety, making it an ideal candidate for the detection of *C. cellulosae*.

### Sensitivity of the UPT-LF assay

**Figure 2:**
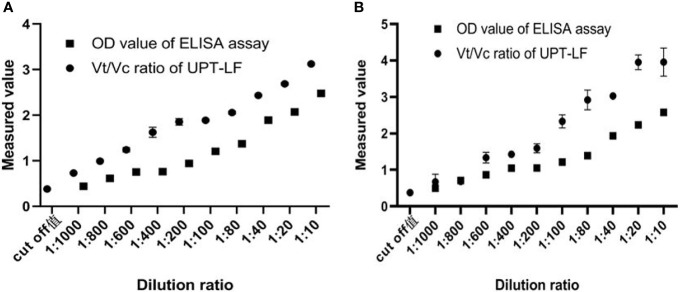
**(A)** TSOL18 and **(B)** GP50, the up-converting phosphor technology-based lateral-flow (UPT-LF) assay is more sensitive than the enzyme-linked immunosorbent assay (ELISA), and the value of Vt/Vc is still higher than that of cut off when diluted 1000 times.

Dilutions of positive serum at a given initial concentration in PBS buffer were detected by UPT-LF assay, and the Vt/Vc value decreased with the increase of the dilution ratio of the tested serum (**Figure 2**). The 1000x dilution was still greater than the UPT-LF assay cutoff value, indicating that the sensitivity of the UPT-LF assay can meet the requirements of rapid detection in the field.

### Specificity of the UPT-LF Assay

**Figure 3:**
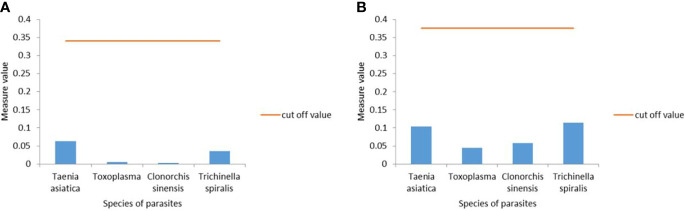
**(A)** TSOL18 and **(B)** GP50, the up-converting phosphor technology-based lateral flow (UPT-LF) assay for detection of *T. asiatica*, *T. gondii*, *C*. *sinensis*, and *T. spiralis*, showed excellent specificity.

This study evaluated the specificity of the UPT-LF test using four enzyme-linked immunosorbent assay (ELISA)-confirmed positive serum samples, including *T. asiatica*, *T. gondii*, *C. sinensis*, and *T. spiralis*, as the study subjects. *C. cellulosae*-positive serum samples served as controls, and the UPT-LF test showed good specificity with all other positive serum samples, producing negative results (**Figure 3**).

### The Stability of the UPT-LF Assay

The sensitivity and specificity of the test strip stored at 4°C for 24 weeks were 100%, indicating that the test strip could be stored at 4°C for at least 6 months, and the sensitivity and specificity of the test strip stored at room temperature were 100% at 16 weeks. There was no significant difference in the color of the detection line or the control line. From the 18th week, the colors of the T line and C line became lighter, so the test strips could be stored for 16 weeks at room temperature. It is proven that this test strip has the characteristics of good stability, strong practicability, easy preservation, and value for market development.

### Comparison of the UPT-LF Assay and ELISA

UPT-LF and ELISA were used to detect 78 negative sera. In the case of TSOL18 and GP50 antigens, 72 were negative for both antigens according to ELISA, and 73 and 76 were negative according to UPT-LF, indicating that UPT-LF and ELISA are consistent in detecting *C. cellulosae*. In the case of TSOL18 and GP50 antigens, the sensitivity of UPT-LF was 93.59% and 97.44%, respectively, while the sensitivity of ELISA was 92.31% for both. Compared to the two methods, the UPT-LF can greatly reduce the false positive rate and can be more sensitive to test samples. Therefore, UPT-LF has the advantages of high speed and easy readability, which makes UPT-LF has broad application prospects.

Specifically speaking, the UPT-LF assay has the following advantages over traditional ELISA. First, the surface-modified UCP particles can be freely combined with a wide range of spectra for quantitative and multiplex analyses; the unique energy conversion process of the UCP particles avoids interference from impurities in the detection background; and the phenomenon of uptransfer luminescence is a physical process using infrared light excitation to produce visible light (Zheng et al., 2015; Huang C. et al., 2019). UPT-LF does not involve chemical reactions, is stable, and does not pose any hazards to laboratory operators or the outside environment. Second, the UPT-LF assay demonstrates good sensitivity and specificity and is suitable for screening and large-scale testing of diseases because it can be performed with a small sample volume within a short period of time (Zhu et al., 2021). Finally, the UPT-LF method is easy to use, portable, and can be performed and analyzed in remote and unprotected areas (Wu et al., 2014; Hua et al., 2015). In addition, the UPT-LF assay requires only a biosensor and test strip, which is less expensive and safer. Overall, the UPT-LF method is suitable for field detection of *C. cellulosae*.

In this study, a preliminary UPT-LF assay for the detection of *C. cellulosae* was developed. This test card uses UCP-labeled TSOL18 or GP50 as the antigen and test strips as the solid-phase vehicle for immunoreactivity. This UPT-LF assay has not only high sensitivity and a wide detection range but also good stability. The strips can be stored at 4°C for six months and at room temperature for four months, indicating that low temperatures are more suitable for storage of the strips and that room temperature or higher temperatures may cause degradation of the antibodies on the conjugated pad or the NC membrane. Using the UPT-LF method, the cystic larvae were significantly distinguished from those of *T. asiatica*, *Toxoplasma*, *C. sinensis*, and *T. spiralis*, indicating high specificity of the test strip.

In conclusion, the UPT-LF assay established in this study provides a safe, reliable, convenient, and rapid method for the quantitative detection of *C. cellulosae*. The preliminary establishment of this diagnostic method will contribute to the further detection of *C. cellulosae* and enhance the screening and diagnosis of *C. cellulosae*, thus contributing to socioeconomic and human health improvement.

## Data Availability Statement

The original contributions presented in the study are included in the article/supplementary material. Further inquiries can be directed to the corresponding authors.

## Ethics Statement

The animal study was reviewed and approved by The University of Jilin Animal Care and Use Committee (IZ-2009-08).

## Author Contributions

SS and XLL conceived and designed the experiments. XW performed the experiments. YC and WS analyzed the data. ML, XC, and XNL. DZ and YQ wrote the manuscript. All authors contributed to the article and approved the submitted version.

## Funding

This work was supported by the National Key Research and Development Program of China (2017YFD0501300); the National Natural Science Foundation of China (NSFC 32160842,31960707, 31460658); the Natural Science Foundation of Inner Mongolia Autonomous Region (2021MS03037).

## Conflict of Interest

The authors declare that the research was conducted in the absence of any commercial or financial relationships that could be construed as a potential conflict of interest.

## Publisher’s Note

All claims expressed in this article are solely those of the authors and do not necessarily represent those of their affiliated organizations, or those of the publisher, the editors and the reviewers. Any product that may be evaluated in this article, or claim that may be made by its manufacturer, is not guaranteed or endorsed by the publisher.
